# Attentional Prioritization of Infant Faces in Parents: The Influence of Parents’ Experiences of Care

**DOI:** 10.3390/ijerph20010527

**Published:** 2022-12-28

**Authors:** Micol Gemignani, Michele Giannotti, Xenia Schmalz, Paola Rigo, Simona De Falco

**Affiliations:** 1 Department of Psychology and Cognitive Science, University of Trento, Corso Bettini 84, 38068 Rovereto, Italy; 2 Department of Child and Adolescent Psychiatry, Psychosomatics and Psychotherapy, University Hospital, LMU Munich, 80336 Munich, Germany; 3 Department of Developmental Psychology and Socialisation, University of Padua, 35131 Padua, Italy

**Keywords:** attention, infant, Go/no-Go task, parenting, attachment, parenting styles, involvement

## Abstract

Infant faces are prioritized by the attentional system in parents, resulting in a greater cognitive engagement in terms of response time. However, many biological, contextual and environmental factors relating to this cognitive mechanism have been left unexplored. To fill this gap, this study aims to (i) confirm that infant faces engage more attention compared to adult faces; (ii) investigate whether the attention to infant faces is affected early care experiences of parents; (iii) explore the effect of parents’ sex by taking the amount of involvement with early childcare into consideration. 51 mothers and 46 fathers completed a modified Go/no-Go task, a brief sociodemographic questionnaire, the short version of the Adult Parental Acceptance–Rejection scale, and an ad-hoc question relating to the amount of parental involvement with early childcare. Parents’ response times were slowed in the presence of infant versus adult faces. Parents whose mother was perceived as more sensitively accepting were more engaged by infant cues. By considering the amount of early parental involvement, the sex of parents did not significantly interact with the type of face. These findings provide new insights on the attention process in response to infant cues in parents and suggest that the investigation of experience-based factors may shed further light on this topic.

## 1. Introduction

Building on John Bowlby and Mary Ainsworth’s principal works [1,2], infant faces are extremely relevant for parents, who need to accurately detect and sensitively respond to infants’ needs. In this regard, the elaboration of infant faces and their expressions has been considered a central mechanism of sensitive parenting [3], which has long-lasting effects on the healthy development of the child [4]. The perception of infants’ cues seems deeply rooted in the human brain [5,6,7]. The “baby schema” characteristics [8,9], such as the configuration of perceptual infantile features (i.e., protruding foreheads, small chins, bulging cheeks, large low-set eyes, narrow noses and small mouths) appear to trigger the activation of brain areas subserving a preparatory response in adults, which might reflect the implicit preparation to respond to infant faces [10]. Furthermore, motivation related brain regions as well as those linked to social cognition and emotions have been found activated in response to infant stimuli [11,12,13]. The face of infants is prioritized by the attentional system of parents [14]. Pearson and colleagues [15] investigated whether pregnant women had an attentional bias toward emotional or non-emotional infant or adult faces by adopting a modified Go/no-Go task. The authors demonstrated that pregnant women took longer to respond to the peripheral stimuli when infant faces, especially those displaying distressed facial expressions, appeared on the central Go/no-Go signal. In a following study [16], a greater attentional bias toward infant distressed faces in pregnant women was significantly linked to more successful mother-infant bonding at 3–6 months postpartum. The influence of parental status on the attention to infant faces has been addressed by Thomson-Booth and colleagues [17], who used a modified irrelevant feature visual search paradigm [18,19]. In general, women showed greater attentional interference (slower response times; RTs) in search arrays containing infant faces versus adult faces as task-irrelevant stimuli. Considering whether the attention process was affected by parental status, first-time mothers had longer RTs to infant compared to adult faces than did non-mothers. In another study [20], infant faces elicited greater attention compared to pre-adolescent, adolescent, or adult faces in mothers versus non-mothers. Oliveira and colleagues [21] investigated the emotional interference of infant and adult faces on the automatic attention in parents and non-parents. In line with previous evidence, parents showed a higher attentional bias toward infant versus adult faces than non-parents. As a result, infant faces seem to be more salient for parents compared to non-parents, but many biological and contextual factors relating to this cognitive mechanism have been left essentially unexplored.

Of note, some experience-based factors, such as the quality of early experiences of care, has been demonstrated to influence the responsiveness of parents to infant cues [22]. Early experiences with their own caregivers guide the development of a relationship model in children, which in turn regulates the interactions with significant others [1]. Following this, remembered maternal rejection was negatively associated with both implicit and explicit responses to infants at the 6th month of pregnancy, and with implicit responses to infants at 3rd month after the childbirth [22]. Despite much evidence assessing the role of attachment representations on adults’ attention to other categories of stimuli, such as unfamiliar or familiar emotional faces of adults e.g., [23,24,25,26], only two studies [27,28], to the best of our knowledge, explored the attentional bias toward infant faces and its relationship with the attachment representations. Notably, both studies included a sample of non-parents. In relation to eye movement data, Jia and collaborators [28] demonstrated that the participants’ patterns of attachment, measured with the Experiences in Close Relationships [29], modulated the attentional bias toward infants. Women with higher attachment avoidance had less attentional bias for infant compared to adult faces for all facial expressions. Long and collaborators [27] found that the attachment tendency to avoidance, measured though the State Adult Attachment Measure [30], negatively predicted individuals’ attentional bias toward infant faces at 500 milliseconds (ms). However, no studies up to date have examined how perceived early care experiences of parents, measured though the Adult Parental Acceptance–Rejection scale [31,32], may have an impact on the attentional bias toward infant faces.

On another side, due to the greater importance that has been placed on motherhood, the investigation of fathers’ characteristics has been usually disregarded. To extent of our knowledge, only one study [21] found an enhanced attention bias to infant versus adult faces, independently of face valence, in both mothers and fathers when compared to non–parents. However, this study included only 11 fathers in the sample, and differences related to parental sex were not highlighted. Overall, enriching the comprehension of sex differences in parents could be compelling. This may reinforce the idea that maternal and paternal roles are convergent in some ways [33] and help clarify the mixed results that have been outlined so far e.g., [21,34,35,36]. Where sex differences have been found in behavioral studies, they have described as small and subtle in nature [34,37]. Relatively few studies have investigated these effects at the neural level, although differences have been detected in the early P1 responses to infant faces in mothers versus fathers [35]. Whilst the P1 response was bilateral in mothers, it was smaller in the left hemisphere versus right hemisphere in fathers [35]. Functional MRI studies have also demonstrated differential responses to hunger cries in medial Default-Mode Network regions; that is, women decreased activity in these regions when they suddenly and passively heard infant hunger cries, but men did not [36]. However, the size of the sample in this study did not allow to have sex comparisons within the parents’ group only [36]. To clarify these complex results, it should be noted that some contextual and experience-based factors, such as the amount of early parental involvement in childcare, may partially explain those effects previously related to parental sex. In different-sex couples, mothers have been consistently found more involved with early childcare when compared to fathers [38,39]; therefore, the effects related to sex and parental involvement with childcare may have overlapped in previous studies [40,41]. In line with recent research on same-sex couples [42], it has been suggested to consider sex and involvement as two independent variables also for different-couples [43]. However, no study to date has investigated parents’ sex differences in the attentional bias to infant cues by considering the amount of early involvement in childcare as a potential confounding factor.

Overall, our study aimed to enrich the understanding of the attentional processing of infant stimuli and to enhance knowledge on how this might vary in relation to parental experiences of care during childhood. Also, we aimed at investigating the effects related to parents’ sex by considering the amount of involvement with early childcare as a potential confounding variable. For these purposes, we first implemented an online task to confirm that baby faces retain more attention compared to adult faces. In line with the abovementioned findings, we expect that infant faces interfere with the task performance more than adult faces, slowing RTs to peripheral stimuli in Go conditions. Secondly, we examined whether the quality of early care experiences during childhood may affect parents’ attention toward infant faces. In line with previous evidence, we hypothesize that parents with warmer parental care experiences would pay more attention to infant compared to adult faces. Thirdly, we explored whether the attention to infant faces could be affected by parents’ sex considering the amount of parental involvement with early childcare. Due to the inconsistent findings highlighted so far about sex differences, we did not test a priori hypothesis. Despite promising pioneering investigations about the importance of parental involvement on the responsiveness to infant cues, we were still reluctant to derive hypotheses based on the results of previous studies.

## 2. Materials and Methods

### 2.1. Participants

114 parents (N = 57 mothers and N = 57 fathers being a couple) were recruited to participate in the study. As inclusion criteria, all parents cohabited with their partner at the time of the experiment, and the age of their (only or youngest) child ranged between 2 and 60 months. Only participants with complete data (N = 103 parents) were included in the analyses. Due to low accuracy level in the behavioral paradigm (i.e., accuracy lower than the chance level in at least one block of the task), 6 participants were excluded from the final sample, which eventually included 97 parents, i.e., 51 mothers (53%) and 46 fathers (47%). The Socio-Economic Status (SES) index, according to Hollingshead’s [44] criteria, showed a variation from low (2%) to medium-low (5.2%), medium (42.3%), medium-high (43.3%), and high SES (7.2%) [45]. An extensive overview of the characteristics of the study participants is reported in Table 1.

### 2.2. Self-Reported Measures

All the measures were administered online using Qualtrics [46]. A brief sociodemographic questionnaire was administered to collect basic information on participants’ age, sex, occupation, level of education, parity, and age of the (youngest) child in months. Participants completed the Italian validated short-form version [31] of the Parental Acceptance-Rejection scale (PARQ) [32]. Parental acceptance has been conceptualized in terms of warmth, affection, care, comfort, concern, nurturance, support, or simply love that children and adults can receive from others. Rejection is defined in terms of the absence of these accepting behaviors, and the presence of a variety of hurtful practices [47]. The Italian validated short-form version of PARQ consists of two scales measuring the experience of maternal and paternal care. Each scale, which has 24 items, originates a total maternal/paternal rejection score consisting of four different dimensions: (1) warmth/affection (e.g., My [mother/father] makes me feel wanted and needed); (2) hostility/aggression (e.g., My [mother/father] treated me harshly); (3) indifference/neglect (e.g., My [mother/father] paid no attention to me as long as I did nothing to bother [her/him]); and (4) undifferentiated rejection (e.g., My [mother/father] saw me as a big nuisance). Participants indicated how well each statement described their experience of early care using a four-point Likert scale (from 4 = almost always true to 1 = almost never true). The amount of early involvement with childcare was computed for each parent using one question extracted and adapted from Abraham and colleagues’ [42] semi-structured interview. Particularly, all parents were asked—how many hours a week do you spend alone with your child?—and the response was provided on a quantitative continuous scale. This item has been translated into Italian and back-translated by a native English speaker. In cases in which parents had multiple children, they were asked to refer to the amount of time spent with the youngest one. We made sure that parents answered that question referring to a period within 3 years of age of their child. The importance of early parental involvement in this developmental stage has been supported by seminal research, proving that it may be related to both children (i.e., attachment security) and parents’ (i.e., paternal sensitivity) characteristics [48,49].

### 2.3. Stimuli and Experimental Task

**Figure 1:**
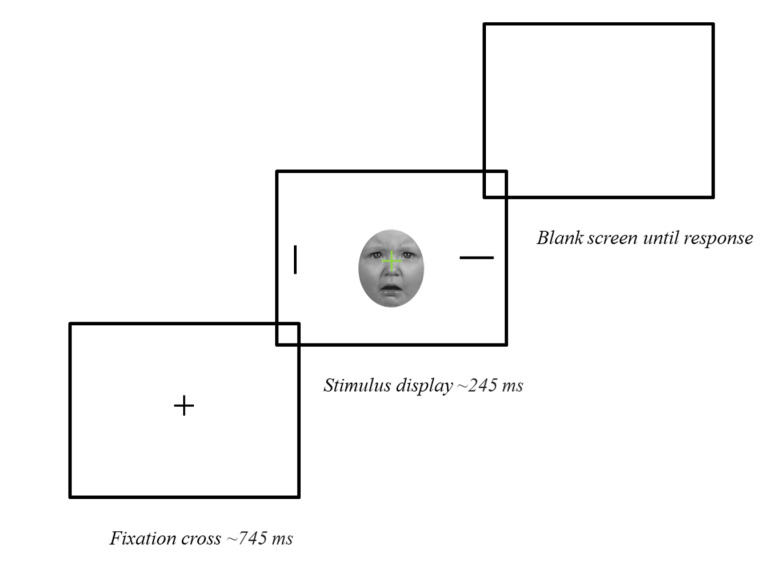
An example of a trial structure and screen presentation sequence. Each trial begins with a central fixation cross (745 ms). The stimulus display (245 ms) consists of an infant or adult face shown behind the central green or red cross, and two-line targets (one horizonal, one vertical) presented peripherally. The screen response (blank screen) is aborted if no response is registered within 2000 ms.

As experimental stimuli, we used 36 images of unfamiliar faces from infants aged 4–12 months (6 males; 6 females) extracted from the Tromso Infant Faces Database (TIF) [50], and 36 images of unfamiliar adult faces (6 males; 6 females) taken from the Karolinska Directed Emotional Faces (KDEF) [51]. Each identity had three standardized images showing a frontally oriented positive (happy), neutral, and negative (sad) facial expression. Of note, infant negative expressions typically showed babies crying. All faces were cropped in an oval shape, converted into grayscale, and presented against a uniform white background. Images were matched for size using GNU Image Manipulation Program v. 2.8.22 [52], and equalized for low-level properties (luminance, saturation) using MATLAB [53]. All the stimuli were 170 × 198 pixels. In terms of the experimental procedure (see Figure 1), we implemented a modified Go/no-Go task adapted from an established paradigm [54], which has been used to measure attentional bias to infant and adult faces e.g., [15,16,21,55]. The experimental task was run on JATOS server [56]. Participants were asked to focus on a central fixation point “+” which signaled the Go/no-Go condition by turning into green or red font, respectively. Simultaneously, two lines, one horizontal and one vertical, appeared at the periphery of the screen. Faces appeared behind the fixation Go/no-Go cross. Only for Go trials, participants were asked to indicate on which side of the screen the vertical line appeared by pressing “n” (for right) or “v” (for left) on the keyboard. Go trials required the disengagement of attention from the central Go signal and the orientation toward the peripheral vertical line. Participants had to withhold a response in no-Go trials. First, participants completed two practice blocks of 12 trials. The first block did not show faces, whereas the second block displayed faces. Experimental trials consisted of 6 blocks of 36 trials (24 Go and 12 no-Go), one for each face valence condition. The order of trials was randomized within blocks, but Go trials occurred twice as frequently as no-Go trials. The experimental conditions were blocked to decrease the potential risk of popout [57], but the block order was randomized across subjects. The target line location was balanced within each block (50% on the right; 50% on the left of the screen). Attention was measured by calculating RTs (in ms) to identify the location of the target vertical line from the onset of the stimulus display, only in Go trials. Faces that recruited greater attention would result in slower RTs in identifying the target vertical line. The rationale of this methodology has been extensively reported in Bindemann and colleagues [54].

### 2.4. Stimuli and Experimental Task

The whole experimental procedure was completed online due to the COVID-19 pandemics restrictions. The procedure was approved by the ethical committee of the University of Trento, in accordance with the declaration of Helsinki. Participants were asked to sign an informed consent, then they were sent a link to complete the self-reported measures. A single code was assigned to each member of the couple, to identify them separately. Next, participants were invited to participate in a Zoom meeting in due course. Both parents completed the experimental task within the same day; whenever it was not feasible, they did the task in separate sessions. Participants were asked to be sitting in a quiet environment while doing the experimental task on their computer. Each participant was accurately instructed to make a localization judgment only for Go trials, by pushing the “n” or “v” whether the vertical line appeared on the right or the left periphery of the screen, respectively. Participants were instructed to keep their left index finger on the “v” and their right index finger on the “n”. Participants were told to ignore any stimuli that might be presented at fixation, and to be as accurate as possible in their responses. After completing the two practice blocks, the experimenter made sure that any doubts would be solved. The experimenter shut down their microphone and camera during the task, and carefully checked the participants’ engagement in completing the whole procedure. In such a way, the experimenter could monitor the quality of data collected.

### 2.5. Data Analysis

Analyses exploring preliminary differences between mothers and fathers were performed through linear mixed-effect models for the dependent variables, including the dyad as a random factor. The models were performed using the lme4 (Version 1.1-28) library [58] in Rstudio (Version 4.1.1) [59]. To facilitate the interpretation of results, we recoded parents’ sex as follows, i.e., =1, “female”; =−1, “male”. Analyses were conducted for evaluating the number of correct answers in Go trials for different blocks. Individuals whose accuracy was below the chance level for at least one block (N = 6 participants) were excluded. The measure focusing on response accuracy was then analyzed using a generalized mixed-effects model to accommodate the binomial nature of the dependent variable. RTs were computed on the time elapsing from stimulus display onset until the response on Go trials. Across all blocks, responses below 100 ms or above 1700 ms were considered outliers and removed. RTs were analyzed via linear mixed-effects models [58]. Only correct trials were considered for these analyses. To improve the normality of data, RTs were log-transformed, and the distribution was checked visually on the trial-, participant- and item- levels. As the distributions were approximately normal, we did not exclude any further items, participants, or trials. To make the slopes more easily interpretable, all conditions of the experimental task were contrast-coded, such that the intercepts reflected the grand mean for all conditions. With respect to the PARQ, the total scores of the paternal scale (parq_father) and maternal scale (parq_mother) were considered separately [22]. Given the scarcity of high PARQ scores among participants, the measures were transposed by computing the inverse scores (i.e., −1/parq_father; −1/parq_mother) to reduce the effect of potential outliers. Missing data for the parq_mother/father (N < 97; see Table 1) were due to the lack of some participants’ experience with their own parents during childhood (e.g., premature death of one parent). Missing data were not replaced. The measures of involvement and child age were centered by subtracting the overall mean across participants. To facilitate the interpretation of results, we dichotomized the number of children as follows, i.e., = 1, “primiparous”; >1, “multiparous”. We performed a robustness check for the models. In Supplementary Material, the main models are marked in bold. Unless explicitly noted, the results from the supplementary analyses converged with those reported in the article. We interpreted effects and interactions as significant after adjusting the α level for multiple comparisons according to Bonferroni correction (*p* < 0.05).

## 3. Results

### 3.1. Preliminary Results

See Table 1 for the descriptive characteristics of the study participants. Linear mixed-effect models displayed significant differences between mothers and fathers. Mothers were significantly younger as compared to their counterparts (β = −3.532, st. err = 0.877, t = −4.024, *p* < 0.01). There were no significant differences in terms of paternal and maternal acceptance/rejection behaviors scores between mothers and fathers. Compared to fathers, mothers were overall more involved with early childcare (β = 16.371, st. err = 3.895, t = 4.203, *p* < 0.01).

**Table 1:** A broad overview of the characteristics of parents. N = number; SD = standard deviation.

Variables		N	Mean or Percentage	SD
Sex		97		
Male		46	47.4%	
Female		51	52.6%	
SES		97	41.454	9.675
Parents’ age		97	35.216	6.67
Child’s months		97	21.67	17.722
Number of children		97		
Nulliparous		57	59%	
Multiparous		40	41%	
PARQ (father)		95	38.695	10.571
PARQ (mother)		96	38.104	10.571
Early involvement with childcare		97	15.825	20.753

### 3.2. Main Analysis

The overall accuracy for Go trials was 96%, which confirmed the ability of participants to complete the task as instructed. At first pass, we fitted a generalized mixed-effect model with face and expression type predicting trial-level accuracy. Due to the high level of accuracy, this analysis did not yield any significant result; thus, all subsequent models used RTs as the dependent variable. In relation to this, 0.3% of the trials were considered outliers and removed (RTs < 100 ms or >1700 ms). In Table 2, the descriptive statistics of the (non-log transformed) RTs as a function of the different conditions, in mothers and fathers, are reported. To investigate the first aim of this study (see Table 3), we implemented a linear mixed-effect model in which face and expression type were used as fixed terms and the interaction was considered. The model included random intercepts for participants and image stimuli. This showed a main effect of face type (β = −0.011, st. err = 0.001, t = −7.032, *p* < 0.01), as infant faces engaged more attention compared to adult faces. The main effect of the expression type approached significance in the initial model (β = 0.003, st. err = 0.002, t = 1.91, *p* = 0.06), but the result did not remain statistically significant after varying the slope of the main effect. The interaction between the fixed terms was not significant. To explore the second aim (see Table 3), the two variables parq_father and parq_mother were considered separately. We performed two different models which indicated an interaction worth following up between the type of face and the early experience with mothers (β = 0.0004, st. err = 0.0001, t = 3.334, *p* < 0.01). The interaction between the type of face and the perceived experience with father in childhood was not significant. Then, we implemented a more parsimonious model to investigate the interaction between the type of face and the parq_mother variable. This confirmed the main effect of face type (β = −0.0286, st. err = 0.005, t = −5.492, *p* < 0.01) and highlighted a two-way interaction between face type and the perceived experience with mother in childhood (β = 0.0004, st. err = 0.0001, t = 3.358, *p* < 0.01). In particular, parents who remembered an experience of maternal acceptance (i.e., a lower score of parq_mother) allocated more attention toward infant versus adult faces. The attentional bias to infant versus adult faces tended to disappear in those parents who were highly rejected by their mothers. The same results were replicated by using the transposed parq_mother variable to check for possible effects of outliers. To explore the third aim, we built a model in which sex and early involvement were added as fixed effects in addition to the type of face, and their interactions with the type of face. Of note, we decided to enter both sex and involvement variables in the same model. Given that there was no significant effect of expression, we collapsed across the expressions. By an iterative reduction of model complexity, we arrived at a parsimonious model [58]. The model confirmed the main effect of face type in the same direction as found before, with higher RTs for infant compared to adult faces (β = −0.014, st. err = 0.002, t = −6.69, *p* < 0.01), and highlighted a two-way interaction between the face type and amount of early parental involvement (β = −0.0004, st. err = 0.0001, t = −2.838, *p* < 0.01). Thus, more involved parents in early care allocated more attention (i.e., slower RTs) toward infant faces relative to adult faces. This model revealed another main effect of sex (β = −0.05, st. err = 0.02, t = 2.343, *p* = 0.02), such that mothers were overall slower in responding to the task compared to fathers regardless of the type of face (See raw RTs data in Table 2). Nonetheless, the effect of sex did not remain significant after performing a Bonferroni correction for the number of effects and interactions in the model [60]. Likewise, a three-way interaction between face type, involvement and sex emerged (β = 0.0003, st. err = 0.0001, t = 2.232, *p* = 0.03), but it did not remain significant after the correction of the α level. Due to their potential confounding effects, we included the child age (of the youngest child) and the dichotomized parity as covariates in all models. In particular, we were interested in controlling the interactions of these factors with the type of face. After controlling for the covariates, the models confirmed all the effects reported before. For an extensive overview of models implemented, see Supplementary Material.

## 4. Discussion

This study investigated the automatic attention to infant and adult faces in a group of mothers and fathers, and whether their care experiences during childhood had an impact on the allocation of attention toward infant faces. Furthermore, we examined the impact of parents’ sex on the attentional bias towards infant cues, by taking the early involvement with childcare into account as a potential confounding factor. To the best of our knowledge, the current study is the first one considering the amount of parental involvement in early childcare when assessing the role of parents’ sex on the attention toward infant faces. 

First, we found that RTs were slower in the presence of infant compared to adult faces. Infant facial cues provide caregivers with a wealth of information about the infants’ state and guide them through the selection of appropriate caretaking behaviors. In terms of brain activations, infant faces provide privileged access to neural mechanisms that ignite motivational states across the whole brain [6]. On this note, the attentional prioritization of infant faces and the difficulty to disengage from them may be a key feature for parenting, forming one of the foundations upon which attachment is built [37]. Our result is in line with strong empirical evidence displaying that parents allocate more attention to infant versus adult faces e.g., [14,15,16,17,20,21]; for this reason, this result may also be interpreted as a methodological check. 

Extending previous findings on non-parents by using a self-reported measure of perceived early parental care, this study demonstrated that experiences of receiving care from one’s own mother during childhood might play a critical role in regulating attentional responses to infant cues. Parents whose mothers were perceived as more sensitively accepting, were more engaged by infant versus adult cues. By contrast, under less optimal circumstances (i.e., perceived maternal hostility, neglect, and rejection), the attentional prioritization of infants may be weakened. Previous studies reported that rejecting experiences of care negatively predicted the amount of attentional bias toward infant faces in non-parents [27,28]. By adopting a neuroscientific perspective, mothers’ insecure attachment has been consistently found to negatively shape neurobiological mechanisms related to processing of infant cues e.g., [61,62]. Strathearn and colleagues [61] demonstrated that mothers with secure versus insecure attachment showed an enhanced activation of reward-related brain regions when they were presented with their own infant’s smiling face. When compared to insecure mothers, they consistently showed a greater activations of reward processing brain regions when presented with own infant’s sad face. In addition, they had an increased peripheral oxytocin response while interacting with their infants, which was positively associated with the activation of oxytocinergic and dopamine-associated reward processing brain regions. Another study addressing the electrocortical activity of the brain [62] found that secure versus insecure mothers were significantly faster to orient to infant cries (N100), structurally encode their own infant’s face (N170), and attend to infant faces (P300). Extending these findings limited on mothers, our results suggest that parents’ experiences of care during childhood might be also related to the attentional bias toward infant cues. Given that it has been extensively shown that mothers with insecure attachment patterns are less likely to establish secure relationships with their children [63,64], investigating the antecedents of potential problems in parent-child relationship by adopting multiple methodologies (i.e., neuroscientific, observational, and cognitive methods) may be of great ecological relevance.

Regarding the investigation of sex differences, the main effect of parents’ sex did not prove stable after the correction for multiple comparisons. One of the most novel aspects of this study relies on the investigation of parental sex differences by considering the amount of early parental involvement as a potential confounding factor in the analyses. As a result, the sex of parents did not interact with the type of face when considering the variability explained by the amount of parental involvement in early childcare. While it is often assumed that women are more emotionally keen to care for infants, little evidence supports this notion with respect to early differences in parental responding [37]. In accordance with this, sex differences between parents detected in previous studies may have partially obscured the effects of some overlapping factors, such as the parental experience with early childcare. As it has been found in the preliminary analyses of this study, in fact, mothers have been described as more involved with early childcare when compared to fathers [38,39]; thus, sex and involvement variables may have shared some sort of variance also in previous studies on different-sex couples. In this regard, our work may be considered as a starting point for future research, which may be encouraged to evaluate the role of parents’ experiences (i.e., early involvement with childcare) when assessing the influence of biological factors on parental responsiveness to infant cues. In an exploratory perspective, it has emerged that more involved parents in early childcare were more engaged, in terms of attention, by infant cues. Although some evidence in pregnant mothers suggested that the attentional engagement with infant faces may be established prenatally and thus partially independent on caregiving experience e.g., [15,16], the enhanced perceptual processing of infant stimuli was found related to early childcare experiences in this study. However, given that the amount of early involvement with childcare was considered as a marginal variable in our work, related results should be interpreted as preliminary.

Of note, this study has not found an interaction effect between the type of face and facial expression, as it has been detected in previous studies e.g., [15,16]. Even though the specific valence of infant cues elicits a corresponding caregiving behavior, baby faces seemed to capture parents’ attention irrespective of the emotional content. In a similar vein, Long and colleagues [27] did not find a moderating effect of facial expressions on attentional bias toward infant faces in non-parents. In a sample of parents, Oliveira and colleagues [21] consistently found that the attentional bias toward infants was independent of the emotional facial expressions. However, given that affective stimuli generally attract more attention than neutral stimuli e.g., [65], the generalizability of our result may be tested in future studies. For example, it would be interesting to see whether in the blocked conditions, as we used here, there may be some adaptation or habituation to the expressions, thus reducing the attentional saliency of the emotions expressed.

## 5. Limitations

The current results should be considered in the context of some limitations. First, the heterogeneity of the sample in terms of child age or parity could have reduced the generalizability of our findings. However, all the main findings proved stable after controlling for the child age and parity as covariates in the analyses. It should be said that we adopted less stringent requirements in terms of child age to also include fathers in our study, considering that they have been underrepresented in parenting research. Secondly, data from parents being in a couple may have shared some sort of dependencies, which might be explored in larger samples by adopting more complex statistical models (APIM) [66]. It is worthy to mention that, to partially overcome this limitation, we accounted for the dyad as a random factor in our preliminary linear mixed-effect models. As a third point, observations of parental responses in a standardized environment, i.e., in a laboratory context, may have increased the validity of the procedure. However, the online version of the task allowed researchers to overcome the restrictions related to COVID-19 pandemic in the Italian context. Moreover, it has been recently demonstrated that, despite not being as precise as the lab-based equivalents, the precision of the web-based tasks is generally reasonable, especially if they include the presentation of visual stimuli [67]. As an important limitation, the total amount of time spent with early childcare in a week may be a poor index of the wide parental childcare experience [40,68]. Also, this question may have been a bit ambiguous for participants, who may have referred to the current week or previous weeks whenever the age of their children was greater than 3 years. However, another recent study [69] found promising evidence concerning the Amygdala connectivity only when the number of hours spent in childcare was considered. Of note, they calculated a mean score for each participant to indicate the amount of direct childcare per week in hours [69]. Notwithstanding this consideration, our finding related to early involvement relies on a very limited index, and thus it should be interpreted with caution.

As a final remark, although we conducted Bonferroni corrections, experiment-wise alpha may still be inflated given the many models tested for a given hypothesis.

## 6. Future Directions

Ecologically, it is important for responsive mothers and fathers to rapidly attend to infant cues in an environment where other information competes for attention. In future research, it is worthwhile to examine whether the variation in attentional processing on a computer test might constitute an early determinant of the quality of real–life interactions [55]. In this regard, presenting mothers with images of their own infants could sound as a more ecological methodology to study the coupling between attentional bias and the quality of caregiving. In addition, to test the validity of the evidence presented here, our task could be tested in a lab-based and more controlled environment. Since our findings about sex were unclear, we suggest further exploring this topic by including other factors related (but not overlapping) to sex or gender (i.e., gender roles and norms) [70]. In addition, the replication of our findings in samples of same-sex parents might help to better disentangle the variance explained by sex and parental involvement [41]. It would be nice, for future research, to provide a more in-depth analysis of the relevant parental involvement items to establish a more reliable measure of this construct in relation to other study variables. Parenting involves many types of behaviors, such as, for instance, feeding and comforting, taking care of appointments, and playing. Since these behaviors differ from each other, engaging in certain types of parental behaviors might contribute to tune parents’ cognitive system and neurobiology in specific ways. To obtain a richer and more accurate understanding of this topic, we encourage future research to consider both quantitative and qualitative aspects of early parental involvement, by adopting a sounder methodology [40], to test possible long-lasting effects on both parents and children.

## 7. Conclusions

Overall, this work represents a critical step forward in indicating the antecedents affecting the automatic attentional bias toward infant cues in mothers and fathers. After controlling for the child age and parity, the main findings confirmed that parents allocated more attention toward infant as compared to adult faces. Parents’ attentional bias toward infant faces was found related to the early experience of one’s own maternal care. Preliminary evidence suggested that the attentional bias toward infant faces may be also related to early parental investment in childcare, rather than to parents’ sex. Even though we acknowledge the limitations of our methodology in assessing parental involvement, this does not imply that there is no merit in investigating the influence of parental involvement on the responsiveness to infant cues. Eventually, this study should be considered as pioneering in trying to distinguish the variability explained by biological (i.e., sex) and experience-dependent factors (i.e., parental involvement) in parenting research.

## Figures and Tables

**Table 2:** Mean (SD) of the non-log transformed RTs as a function of the different conditions in mothers and fathers.

Conditions	Mothers (N = 51)	Fathers (N = 46)
Adult positive	687.4 (145.9)	619.9 (128.7)
Adult neutral	685.1 (148.0)	621.5 (117.8)
Adult negative	681.7 (154.8)	623.7 (139.9)
Infant positive	705.5 (162.9)	638.5 (128.4)
Infant neutral	709.2 (168.9)	629.0 (125.8)
Infant negative	702.4 (165.6)	628.3 (125.9)

**Table 3:** An overview of the aims, independent variables, random effect structures, and significant results of the main analyses of the study.

Aim of the Model	Independent Variables of the Model	Random Effect Structures	Significant Main Effects or Interactions
Aim 1: investigate whether baby faces retain more attention compared to adult faces	Face type, expression type, and their interaction	Subject, image	Main effect of face type: infant faces engage more attention compared to adult faces.
Aim 2: investigate whether the quality of parents’ experiences of care during childhood affects the attentional bias to infant faces	Face type, the interaction between face type and early maternal care	Subject, image	Main effect of face type: infant faces engage more attention compared to adult faces. Two-way interaction between face type and the maternal early care experiences: those parents who remember an experience of maternal acceptance allocate more attention toward infant faces.
Aim 3: investigate whether the attentional bias is affected by parents’ sex by considering the early parental involvement with childcare	Face type, parents’ sex, early involvement, and their interactions with face type	Subject, image	Main effect of face type: infant faces engage more attention compared to adult faces. Two-way interaction between face type and early parental involvement: more involved parents with early childcare allocate more attention toward infant faces relative to adult faces.

## Data Availability

The data presented in this study are available on request from the corresponding author. The data are not publicly available due to privacy restrictions.

## Supplementary Materials

The following supporting information can be downloaded at: https://www.mdpi.com/article/10.3390/ijerph20010527/s1.
