# Supplementary material for: Attentional Prioritization of Infant Faces in Parents: The Influence of Parents’ Experiences of Care
Source: Int J Environ Res Public Health. 2022 Dec 28;20(1):527. doi: 10.3390/ijerph20010527 (PMC9819530; doi:10.3390/ijerph20010527)
Supplement: Supplementary file 1 [file ijerph-20-00527-s001.zip › ijerph-2012009-SM.pdf]

This document is R code. Please, contact the corresponding author to have a high-level description for non-R users.

Below, the linear mixed-effect models to test preliminary differences between mothers and fathers:

m_parent_age <- lmer(parent_age~sex + (1|dyad), data=part_chars1)

m_parq_father <- lmer(parq_father~sex + (1|dyad), data=part_chars1)

m_parq_mother <- lmer(parq_mother~sex + (1|dyad), data=part_chars1)

m_inv <- lmer(involvement_last~sex + (1|dyad), data=part_chars1)

Only the first and fourth models provided a significant main effect of sex of parents.

Below, the generalized mixed-effects model to test the response accuracy:

model_glmer <- glmer(correct~face_n*expression_n + (1|subject) + (1|image_face), data=df, family="binomial")

Below, the linear mixed-effect model to test the first hypothesis of our study:

m_base <- lmer(logRT~face_n*expression_n + (1|subject) + (1|image_face), data=db)

Other models have been implemented to test the robustness of the results obtained:

m_slope <- lmer(logRT~face_n*expression_n + (1+face_n*expression_n|subject) + (1|image_face), data=db)

m_slope2 <- lmer(logRT~face_n*expression_n + (1+expression_n|subject) + (1+face_n|subject) +(1|image_face), data=db)

m_slope3 <- lmer(logRT~face_n*expression_n + (1+expression_n|subject) +(1|image_face), data=db)

m_slope4 <- lmer(logRT~face_n*expression_n + (1+face_n|subject) +(1|image_face), data=db)

All the models confirmed the main effect of face type. Regarding the effect of the expression type, the results did not prove statistically significant.

Below, the linear mixed-effect models to preliminarily test the second aim:

model_1 <- lmer (logRT~expression_n + parq_mother + face_n + expression_n:parq_mother + face_n:parq_mother + (1|subject) + (1|image_face), data=db)

model_2 <- lmer (logRT~expression_n + parq_father + face_n + expression_n:parq_father + face_n:parq_father + (1|subject) + (1|image_face), data=db)

To further investigate the interaction between the type of face and the early care experiences with mothers, we reduced the complexity of the model as follows:

model_parq_mother <- lmer(logRT~face_n+ face_n:parq_mother+ (1|subject) + (1|image_face), data=db)

To rule out possible effects of outliers, we used the transposed parq_mother variable as follows:

model_parq_mother_inv <- lmer(logRT~face_n+ face_n:inv_parq_m + (1|subject) + (1|image_face), data=db)

This model confirmed the significant interaction between the face type and parq_mother.

Below, the linear mixed-effect model to test the third aim of our study:

model_mod_min_face <- lmer(logRT~expression_n + face_n*sex*involvement_last_c + (1|subject) + (1|image_face), data=db)

Given that no effects have been found about the expression type, the results of the model below were eventually reported:

model_mod_min_face1 <- lmer(logRT~ face_n*sex*involvement_last_c + (1|subject) + (1|image_face), data=db)

To test the effects of possible covariates (child age, parity), all the models marked in bold were further tested after adding the covariates. All the results reported in the manuscript proved stable.
